# Crystal structure of 3-[(*E*)-(2-hy­droxy-3-meth­oxy­benzyl­idene)amino]-1-methyl-1-phenyl­thio­urea

**DOI:** 10.1107/S2056989016005326

**Published:** 2016-04-05

**Authors:** Rajeswari Gangadharan, Mathiyan Muralisankar, Anandaram Sreekanth, Abdullakutty Anees Rahman, K. Sethusankar

**Affiliations:** a Department of Physics, Ethiraj College for Women (Autonomous), Chennai 600 008, India; b Department of Chemistry, National Institute of Technology, Tiruchirappalli 620 015, India; c Department of Physics, RKM Vivekananda College (Autonomous), Chennai 600 004, India

**Keywords:** crystal structure, hydrazinecarbo­thio­amide, thio­urea derivatives, α-*N*-heterocyclic, hydrogen bonding

## Abstract

In the asymmetric unit of the title compound, C_16_H_17_N_3_O_2_S, there are two independent mol­ecules (*A* and *B*), which show an *E* conformation with respect to the C=N bond. An intra­molecular O—H⋯N hydrogen bond with an *S*(6) motif stabilizes the mol­ecular structure. The terminal phenyl and benzene rings are almost orthogonal to each other, the dihedral angle being 87.47 (13)° for mol­ecule *A* and 89.86 (17)° for mol­ecule *B*. In the crystal, weak bifurcated N—H⋯(O,O) hydrogen bonds link the two independent mol­ecules, forming a supra­molecular chain with a *C* ^2^_1_(14)[*R* ^2^_1_(5)] motif along the *b* axis. A weak C—H⋯O inter­action is also observed in the chain.

## Abstract 2

The asymmetric unit comprises two independent mol­ecules. In the crystal, the two independent mol­ecules are linked by bifurcated N—H⋯O hydrogen bonds, forming a supra­molecular chain with a 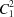
(14)[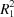
(5)] motif.

## Chemical context   

Thio­semicarbazones have emerged as an important class of S- and N-containing ligands due to their propensity to react with a wide range of metals (Casas *et al.*, 2000 ▸) and their broad spectrum of chemotherapeutic properties (Quiroga *et al.*, 1998 ▸). Their structural diversity is due to their variable coord­inative abilities (Sreekanth *et al.*, 2004 ▸), arising from thio­amido–thio­iminol tautomerism. Thio­semicarbazones usually act as chelating ligands for metal ions through sulfur (=S) and azo­methane (=N—) groups, though in some cases they behave as monodentate ligands through the sulfur (=S) only. They are also important inter­mediates for obtaining heterocylic rings such as thia­zolidones, oxa­diazo­les, pyrazolidones and thia­diazo­les (Greenbaum *et al.*, 2004 ▸; Küçükgüzel *et al.*, 2006 ▸). As a result of their long chain structure, they are very flexible and form linkages with a variety of metal ions. They have also been used for the analysis of metals and in device applications related to telecommunications, optical computing and optical information processing (Tian *et al.*, 1997 ▸).
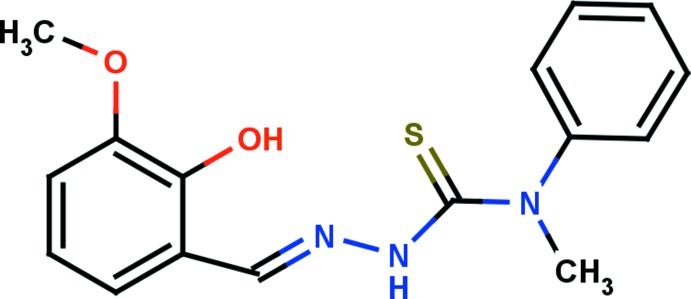


## Structural commentary   

**Figure 1:**
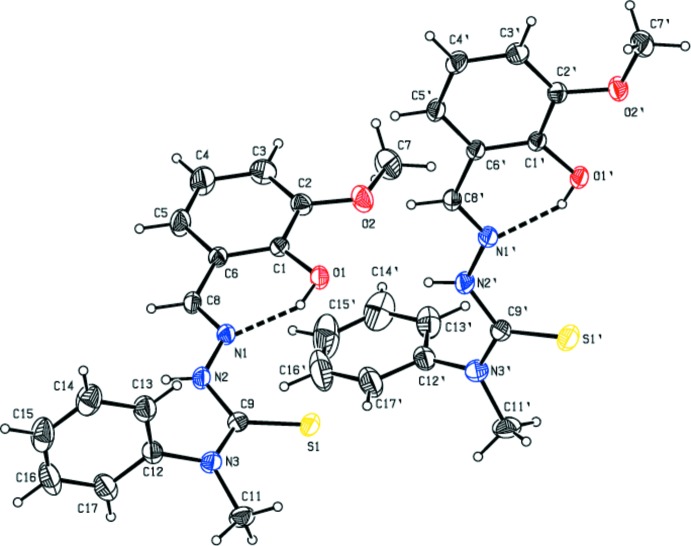
The two independent mol­ecules (*A* and *B*) of the title compound, with atom labelling. Displacement ellipsoids are drawn at the 30% probability level. Dashed lines indicate the intra­molecular O—H⋯N inter­actions.

The asymmetric unit of the compound comprises two independent mol­ecules (*A* and *B*) with almost identical conformations. The hydrazine carbo­thio­amide backbone is nearly planar with a maximum deviation of 0.023 (2) Å at atom N2 for mol­ecule *A* and of 0.054 (2) Å at atom N2′ for *B*. The closeness of the C=S bond lengths [C9—S1 = 1.666 (2) Å and C9′—S1′ = 1.657 (2) Å] to the expected distance (1.60 Å; Allen *et al.*, 1987 ▸; Seena *et al.*, 2008 ▸) indicates that the compound exists in the thione form. This is further confirmed by the N—N and N—C bond lengths (Gangadharan *et al.*, 2015 ▸). The bond lengths in the N—C(=S)—N fragments indicate π delocalization due to the fact that the C—N and C—S bonds are shorter than typical single bonds (*ca* 1.47 and 1.73 Å, respectively) and longer than corresponding double bonds (*ca* 1.29 and 1.55 Å, respectively; Casas *et al.*, 2000 ▸; Tenório *et al.* 2005 ▸). The terminal phenyl and benzene rings are almost orthogonal to each other, with a dihedral angle of 87.47 (13)° for *A* and 89.86 (17)° for *B*. In each mol­ecule (*A* and *B*), an intra­molecular O—H⋯N inter­action (Table 1 ▸) with an *S*(6) ring motif stabilizes the mol­ecular structure (Fig. 1 ▸).

## Supra­molecular features   

**Figure 2:**
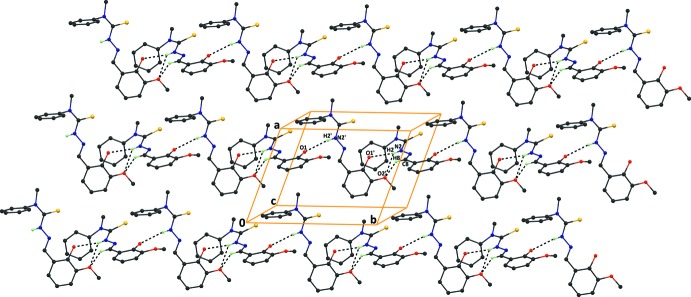
A packing diagram of the compound viewed along the *c* axis, showing the N—H⋯O and C—H⋯O hydrogen bonds (dashed lines). H atoms not involved in the hydrogen bonds have been omitted for clarity.

In the crystal, inter­molecular bifurcated hydrogen bonds (N2—H2⋯O1′^i^, N2—H2⋯O2′^i^, N2′—H2′⋯O1 and N2′—H2′⋯O2; symmetry code: (i) *x*, −1 + *y*, *z*] with 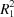
(5) ring motifs inter­link adjacent independent mol­ecules, resulting in a supra­molecular chain with a 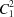
(14)[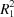
(5)] motif along the *b* axis. An inter­molecular C—H⋯O inter­action is also observed within the chain (Fig. 2 ▸).

## Database survey   

A search of Cambridge Structural Database (Version 5.36; last updated Nov. 2014; Groom & Allen, 2014 ▸) showed three closely related structures with pyridine-2-carbaldehyde thio­semicarbazones, differing from the title compound only in the presence of one or more pyridyl groups instead of the substituted phenyl group. Two of these, namely, (*E*)-4-methyl-4-phenyl-1-(2-pyridyl­methyl­ene)-3-thio­semicarbazide (Rapheal *et al.*, 2007 ▸) and di-2-pyridyl ketone 4-methyl-4-phenyl­thosemicarbazone (Philip *et al.*, 2004 ▸) crystallize in the same *P* 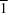 space group of the title compound. The third compound, 2-benzoyl pyridine-*N*-methyl-*N*-phenyl­thio­semicarbazone, crystallizes in *P*2_1_/n. The similarity in bond lengths along the hydrazine carbo­thio­amide moieties and shortening of the C—N single bonds from the normal value (*ca* 1.48 Å) indicate some degree of delocalization in the compounds. The C=S bond lengths in all compared compounds lie in the range 1.66–1.67 Å, inter­mediate between S—C*sp* ^2^ and S=C*sp* ^2^ bond lengths (*ca* 1.75 and 1.59 Å, respectively), showing a partial double-bond character. Similar bond lengths for the C=S bond have also been observed in hydrazine carbo­thio­amide derivatives (Gangadharan *et al.*, 2014 ▸, 2015 ▸; Vimala *et al.*, 2014 ▸). The partial double-bond nature of the C=S bond is a feature in the compared hydrazine carbo­thio­amide derivatives, irrespective of the substituents.

## Synthesis and crystallization   

1.81 g (0.01 mol) of *N*-methyl-*N*-phenyl­hydrazine carbo­thio­amide was dissolved in 20 ml of hot methanol and to this was added 1.52 g (0.01 mol) of 2-hy­droxy-3-meth­oxy­benzaldehyde in 10 ml of ethanol over a period of 10 min with continuous stirring. The reaction mixture was refluxed for 2 h and allowed to cool whereby a shining yellow compound began to separate. This was filtered and washed thoroughly with ethanol and then dried in vacuum. The compound was recrystallized from a hot ethanol solution, giving colourless block-like crystals (yield 91%). Single crystals suitable for X-ray diffraction were prepared by slow evaporation of an ethanol solution at room temperature.

## Refinement   

Crystal data, data collection and structure refinement details are summarized in Table 2 ▸. H atoms were localized in a difference-Fourier map. H atoms bound to O and N atoms were refined freely; refined distances O—H = 0.79 (3) and 0.87 (3) Å, and N—H = 0.80 (2) and 0.83 (2) Å. C-bound H atoms were treated as riding, with C—H = 0.93 or 0.96 Å, and with *U*_iso_(H) = 1.2*U*_eq_(C) for aromatic and 1.5*U*_eq_(C) for methyl groups. The rotation angles for methyl groups were optimized.

## Supplementary Material

Crystal structure: contains datablock(s) I, global. DOI: 10.1107/S2056989016005326/is5446sup1.cif

Structure factors: contains datablock(s) I. DOI: 10.1107/S2056989016005326/is5446Isup2.hkl

Supporting information file. DOI: 10.1107/S2056989016005326/is5446Isup3.cml

CCDC reference: 1435114

Additional supporting information:  crystallographic information; 3D view; checkCIF report

## Figures and Tables

**Table 1:** Hydrogen-bond geometry (Å, °)

*D*—H⋯*A*	*D*—H	H⋯*A*	*D*⋯*A*	*D*—H⋯*A*
O1—H1⋯N1	0.87 (2)	1.85 (3)	2.611 (2)	146 (3)
O1′—H1′⋯N1′	0.80 (3)	1.89 (2)	2.609 (2)	149 (3)
N2—H2⋯O1′^i^	0.80 (2)	2.59 (2)	3.341 (2)	157 (2)
N2—H2⋯O2′^i^	0.80 (2)	2.52 (2)	3.081 (3)	128 (2)
N2′—H2′⋯O1	0.83 (2)	2.51 (2)	3.282 (3)	156.1 (19)
N2′—H2′⋯O2	0.83 (2)	2.62 (2)	3.214 (3)	130.0 (19)
C8—H8⋯O2′^i^	0.93	2.52	3.085 (3)	120

Symmetry code: (i) 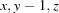.

**Table 2:** Experimental details

Crystal data	
Chemical formula	C_16_H_17_N_3_O_2_S
*M*_r_	315.39
Crystal system, space group	Triclinic, *P* 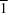
Temperature (K)	296
*a*, *b*, *c* (Å)	9.6869 (2), 12.6140 (2), 14.7498 (3)
α, β, γ (°)	77.839 (1), 76.5330 (9), 70.875 (1)
*V* (Å^3^)	1638.19 (5)
*Z*	4
Radiation type	Mo *K*α
μ (mm^−1^)	0.21
Crystal size (mm)	0.35 × 0.30 × 0.25

Data collection	
Diffractometer	Bruker APEXII CCD
Absorption correction	Multi-scan (*SADABS*; Bruker, 2008 ▸)
*T*_min_, *T*_max_	0.931, 0.950
No. of measured, independent and observed [*I* > 2σ(*I*)] reflections	24246, 6781, 5047
*R*_int_	0.026
(sin θ/λ)_max_ (Å^−1^)	0.628

Refinement	
*R*[*F* ^2^ > 2σ(*F* ^2^)], *wR*(*F* ^2^), *S*	0.047, 0.139, 1.05
No. of reflections	6781
No. of parameters	417
H-atom treatment	H atoms treated by a mixture of independent and constrained refinement
Δρ_max_, Δρ_min_ (e Å^−3^)	0.30, −0.30

Computer programs: *APEX2* and *SAINT* (Bruker, 2008 ▸), *SHELXS97* and *SHELXL97* (Sheldrick, 2008 ▸), *ORTEP-3 for Windows* (Farrugia, 2012 ▸), *Mercury* (Macrae *et al.*, 2008 ▸) and *PLATON* (Spek, 2009 ▸).

## supplementary crystallographic information

### Crystal data

**Table:**

C_16_H_17_N_3_O_2_S	*Z* = 4
*M**_r_* = 315.39	*F*(000) = 664
Triclinic, *P*1	*D*_x_ = 1.279 Mg m^−^^3^
Hall symbol: -P 1	Mo *K*α radiation, λ = 0.71073 Å
*a* = 9.6869 (2) Å	Cell parameters from 6781 reflections
*b* = 12.6140 (2) Å	θ = 1.4–26.5°
*c* = 14.7498 (3) Å	µ = 0.21 mm^−^^1^
α = 77.839 (1)°	*T* = 296 K
β = 76.5330 (9)°	Block, colourless
γ = 70.875 (1)°	0.35 × 0.30 × 0.25 mm
*V* = 1638.19 (5) Å^3^	

### Data collection

**Table:**

Bruker APEXII CCD diffractometer	6781 independent reflections
Radiation source: fine-focus sealed tube	5047 reflections with *I* > 2σ(*I*)
Graphite monochromator	*R*_int_ = 0.026
ω & φ scans	θ_max_ = 26.5°, θ_min_ = 1.4°
Absorption correction: multi-scan (*SADABS*; Bruker, 2008)	*h* = −12→12
*T*_min_ = 0.931, *T*_max_ = 0.950	*k* = −15→15
24246 measured reflections	*l* = −18→18

### Refinement

**Table:**

Refinement on *F*^2^	Primary atom site location: structure-invariant direct methods
Least-squares matrix: full	Secondary atom site location: difference Fourier map
*R*[*F*^2^ > 2σ(*F*^2^)] = 0.047	Hydrogen site location: inferred from neighbouring sites
*wR*(*F*^2^) = 0.139	H atoms treated by a mixture of independent and constrained refinement
*S* = 1.05	*w* = 1/[σ^2^(*F*_o_^2^) + (0.0643*P*)^2^ + 0.4596*P*] where *P* = (*F*_o_^2^ + 2*F*_c_^2^)/3
6781 reflections	(Δ/σ)_max_ = 0.01
417 parameters	Δρ_max_ = 0.30 e Å^−^^3^
0 restraints	Δρ_min_ = −0.30 e Å^−^^3^

### Special details

**Table:**

Geometry. All esds (except the esd in the dihedral angle between two l.s. planes) are estimated using the full covariance matrix. The cell esds are taken into account individually in the estimation of esds in distances, angles and torsion angles; correlations between esds in cell parameters are only used when they are defined by crystal symmetry. An approximate (isotropic) treatment of cell esds is used for estimating esds involving l.s. planes.
Refinement. Refinement of F^2^ against ALL reflections. The weighted R-factor wR and goodness of fit S are based on F^2^, conventional R-factors R are based on F, with F set to zero for negative F^2^. The threshold expression of F^2^ > 2sigma(F^2^) is used only for calculating R-factors(gt) etc. and is not relevant to the choice of reflections for refinement. R-factors based on F^2^ are statistically about twice as large as those based on F, and R- factors based on ALL data will be even larger.

### Fractional atomic coordinates and isotropic or equivalent isotropic displacement parameters (Å^2^)

**Table:**

	*x*	*y*	*z*	*U*_iso_*/*U*_eq_	
C1'	0.52668 (19)	0.69280 (15)	0.27874 (12)	0.0439 (4)	
C2'	0.4045 (2)	0.78361 (16)	0.30538 (13)	0.0490 (4)	
C3'	0.2620 (2)	0.77780 (19)	0.31481 (15)	0.0601 (5)	
H3'	0.1812	0.8388	0.3322	0.072*	
C4'	0.2396 (2)	0.6813 (2)	0.29845 (17)	0.0648 (6)	
H4'	0.1434	0.6777	0.3045	0.078*	
C5'	0.3575 (2)	0.59107 (18)	0.27348 (15)	0.0573 (5)	
H5'	0.3409	0.5262	0.2636	0.069*	
C6'	0.50335 (19)	0.59539 (15)	0.26260 (13)	0.0452 (4)	
C7'	0.3237 (3)	0.9658 (2)	0.3546 (2)	0.0872 (8)	
H7'1	0.2570	0.9977	0.3103	0.131*	
H7'2	0.3639	1.0227	0.3627	0.131*	
H7'3	0.2709	0.9393	0.4140	0.131*	
C8'	0.6264 (2)	0.49832 (17)	0.23490 (14)	0.0520 (5)	
H8'	0.6081	0.4347	0.2237	0.062*	
C9'	1.0115 (2)	0.39863 (17)	0.21247 (16)	0.0578 (5)	
N3'	1.11972 (19)	0.31302 (16)	0.17464 (16)	0.0719 (5)	
C11'	1.2715 (3)	0.2825 (2)	0.1928 (2)	0.0885 (8)	
H11A	1.3240	0.3305	0.1493	0.133*	
H11B	1.3216	0.2048	0.1847	0.133*	
H11C	1.2683	0.2923	0.2561	0.133*	
C12'	1.0977 (2)	0.25550 (19)	0.1077 (2)	0.0702 (6)	
C13'	1.0959 (4)	0.3065 (3)	0.0157 (3)	0.0978 (9)	
H13'	1.1060	0.3792	−0.0019	0.117*	
C14'	1.0793 (4)	0.2515 (4)	−0.0510 (3)	0.1310 (14)	
H14'	1.0777	0.2863	−0.1131	0.157*	
C15'	1.0655 (5)	0.1448 (5)	−0.0232 (5)	0.162 (2)	
H15'	1.0570	0.1057	−0.0675	0.194*	
C16'	1.0638 (6)	0.0947 (4)	0.0671 (5)	0.158 (2)	
H16'	1.0513	0.0227	0.0845	0.189*	
C17'	1.0805 (3)	0.1495 (2)	0.1346 (3)	0.1067 (11)	
H17'	1.0799	0.1146	0.1968	0.128*	
N1'	0.75880 (17)	0.50090 (14)	0.22614 (13)	0.0557 (4)	
N2'	0.87327 (19)	0.40950 (16)	0.19792 (15)	0.0643 (5)	
O1'	0.66301 (16)	0.70549 (13)	0.27051 (11)	0.0575 (4)	
O2'	0.44044 (16)	0.87408 (12)	0.32031 (12)	0.0663 (4)	
S1'	1.04243 (7)	0.48414 (6)	0.27171 (5)	0.0765 (2)	
C1	0.67203 (19)	0.19526 (15)	0.12355 (13)	0.0455 (4)	
C2	0.6581 (2)	0.28011 (17)	0.04616 (15)	0.0573 (5)	
C3	0.6084 (3)	0.2679 (2)	−0.02991 (17)	0.0795 (7)	
H3	0.5993	0.3251	−0.0814	0.095*	
C4	0.5719 (4)	0.1714 (3)	−0.03010 (19)	0.0966 (10)	
H4	0.5370	0.1638	−0.0813	0.116*	
C5	0.5870 (3)	0.0870 (2)	0.04473 (17)	0.0808 (8)	
H5	0.5625	0.0219	0.0439	0.097*	
C6	0.6382 (2)	0.09610 (16)	0.12254 (13)	0.0495 (4)	
C7	0.7106 (4)	0.4531 (3)	−0.0295 (2)	0.1088 (11)	
H7A	0.7793	0.4152	−0.0793	0.163*	
H7B	0.7463	0.5101	−0.0169	0.163*	
H7C	0.6154	0.4881	−0.0483	0.163*	
C8	0.6577 (2)	0.00218 (17)	0.19840 (14)	0.0533 (5)	
H8	0.6352	−0.0631	0.1951	0.064*	
C9	0.8143 (2)	−0.08626 (16)	0.40086 (13)	0.0493 (4)	
N3	0.8251 (2)	−0.17535 (15)	0.47095 (12)	0.0591 (4)	
C11	0.9338 (3)	−0.2017 (2)	0.53275 (16)	0.0704 (6)	
H11D	1.0322	−0.2226	0.4965	0.106*	
H11E	0.9206	−0.2635	0.5811	0.106*	
H11F	0.9198	−0.1362	0.5612	0.106*	
C12	0.7256 (3)	−0.24421 (18)	0.49148 (14)	0.0591 (5)	
C13	0.5790 (3)	−0.1993 (2)	0.52928 (18)	0.0753 (6)	
H13	0.5438	−0.1241	0.5397	0.090*	
C14	0.4834 (4)	−0.2660 (3)	0.5520 (2)	0.0967 (9)	
H14	0.3839	−0.2360	0.5775	0.116*	
C15	0.5371 (5)	−0.3757 (3)	0.5364 (2)	0.1063 (11)	
H15	0.4732	−0.4205	0.5510	0.128*	
C16	0.6833 (5)	−0.4217 (3)	0.4998 (2)	0.1086 (11)	
H16	0.7183	−0.4973	0.4906	0.130*	
C17	0.7792 (4)	−0.3553 (2)	0.47636 (19)	0.0831 (7)	
H17	0.8785	−0.3856	0.4507	0.100*	
N1	0.70532 (18)	0.00886 (13)	0.26945 (11)	0.0508 (4)	
N2	0.7257 (2)	−0.08131 (15)	0.33979 (13)	0.0587 (5)	
O1	0.72154 (17)	0.21260 (13)	0.19595 (10)	0.0583 (4)	
O2	0.6966 (2)	0.37270 (13)	0.05338 (12)	0.0787 (5)	
S1	0.90153 (6)	0.01123 (5)	0.38868 (4)	0.06229 (17)	
H2	0.696 (2)	−0.1333 (19)	0.3390 (15)	0.055 (6)*	
H2'	0.852 (2)	0.3591 (18)	0.1810 (15)	0.053 (6)*	
H1	0.734 (3)	0.151 (2)	0.2363 (18)	0.074 (8)*	
H1'	0.721 (3)	0.646 (2)	0.2595 (18)	0.080 (9)*	

### Atomic displacement parameters (Å^2^)

**Table:**

	*U*^11^	*U*^22^	*U*^33^	*U*^12^	*U*^13^	*U*^23^
C1'	0.0439 (9)	0.0485 (10)	0.0425 (9)	−0.0191 (8)	−0.0086 (7)	−0.0034 (8)
C2'	0.0498 (10)	0.0491 (10)	0.0491 (10)	−0.0173 (8)	−0.0059 (8)	−0.0082 (8)
C3'	0.0463 (10)	0.0629 (13)	0.0674 (13)	−0.0134 (9)	−0.0037 (9)	−0.0127 (10)
C4'	0.0432 (10)	0.0735 (14)	0.0822 (15)	−0.0233 (10)	−0.0069 (10)	−0.0155 (12)
C5'	0.0525 (11)	0.0580 (12)	0.0698 (13)	−0.0256 (9)	−0.0100 (9)	−0.0132 (10)
C6'	0.0442 (9)	0.0475 (10)	0.0466 (10)	−0.0171 (8)	−0.0091 (7)	−0.0057 (8)
C7'	0.0737 (16)	0.0634 (15)	0.127 (2)	−0.0094 (12)	−0.0116 (15)	−0.0429 (16)
C8'	0.0524 (11)	0.0481 (11)	0.0612 (12)	−0.0190 (8)	−0.0109 (9)	−0.0117 (9)
C9'	0.0487 (10)	0.0529 (11)	0.0721 (13)	−0.0172 (9)	−0.0114 (9)	−0.0053 (10)
N3'	0.0459 (9)	0.0640 (12)	0.1040 (15)	−0.0094 (8)	−0.0122 (9)	−0.0202 (11)
C11'	0.0482 (12)	0.0846 (18)	0.121 (2)	−0.0086 (12)	−0.0211 (13)	0.0003 (16)
C12'	0.0456 (11)	0.0557 (13)	0.104 (2)	−0.0131 (9)	0.0044 (11)	−0.0232 (13)
C13'	0.098 (2)	0.092 (2)	0.112 (3)	−0.0441 (17)	0.0020 (18)	−0.0316 (19)
C14'	0.115 (3)	0.180 (4)	0.115 (3)	−0.066 (3)	0.023 (2)	−0.068 (3)
C15'	0.127 (3)	0.165 (5)	0.223 (6)	−0.067 (4)	0.045 (4)	−0.138 (5)
C16'	0.140 (4)	0.096 (3)	0.254 (6)	−0.060 (3)	0.012 (4)	−0.074 (4)
C17'	0.091 (2)	0.0644 (17)	0.162 (3)	−0.0304 (15)	−0.004 (2)	−0.0195 (19)
N1'	0.0465 (9)	0.0501 (9)	0.0722 (11)	−0.0117 (7)	−0.0096 (8)	−0.0177 (8)
N2'	0.0476 (9)	0.0545 (11)	0.0977 (15)	−0.0111 (8)	−0.0134 (9)	−0.0313 (10)
O1'	0.0447 (7)	0.0542 (9)	0.0810 (10)	−0.0192 (7)	−0.0095 (7)	−0.0199 (8)
O2'	0.0575 (8)	0.0539 (8)	0.0921 (11)	−0.0178 (7)	−0.0036 (7)	−0.0287 (8)
S1'	0.0668 (4)	0.0854 (4)	0.0921 (5)	−0.0325 (3)	−0.0188 (3)	−0.0231 (4)
C1	0.0426 (9)	0.0468 (10)	0.0481 (10)	−0.0140 (8)	−0.0082 (7)	−0.0071 (8)
C2	0.0596 (12)	0.0509 (11)	0.0590 (12)	−0.0197 (9)	−0.0097 (9)	0.0019 (9)
C3	0.1032 (19)	0.0799 (17)	0.0563 (13)	−0.0331 (15)	−0.0295 (13)	0.0157 (12)
C4	0.153 (3)	0.093 (2)	0.0704 (16)	−0.054 (2)	−0.0632 (18)	0.0097 (15)
C5	0.124 (2)	0.0737 (15)	0.0705 (15)	−0.0495 (15)	−0.0499 (15)	0.0029 (12)
C6	0.0561 (10)	0.0491 (10)	0.0487 (10)	−0.0187 (8)	−0.0170 (8)	−0.0051 (8)
C7	0.134 (3)	0.085 (2)	0.113 (2)	−0.064 (2)	−0.033 (2)	0.0385 (18)
C8	0.0627 (12)	0.0490 (11)	0.0581 (12)	−0.0254 (9)	−0.0186 (9)	−0.0058 (9)
C9	0.0495 (10)	0.0510 (11)	0.0487 (10)	−0.0139 (8)	−0.0130 (8)	−0.0062 (8)
N3	0.0698 (11)	0.0592 (10)	0.0550 (10)	−0.0263 (9)	−0.0256 (8)	0.0056 (8)
C11	0.0703 (14)	0.0764 (15)	0.0650 (14)	−0.0174 (12)	−0.0310 (11)	0.0032 (12)
C12	0.0838 (15)	0.0537 (12)	0.0465 (11)	−0.0282 (11)	−0.0224 (10)	0.0035 (9)
C13	0.0834 (17)	0.0713 (15)	0.0777 (16)	−0.0339 (13)	−0.0150 (13)	−0.0054 (13)
C14	0.103 (2)	0.116 (3)	0.0865 (19)	−0.064 (2)	−0.0128 (16)	−0.0007 (18)
C15	0.157 (3)	0.108 (3)	0.085 (2)	−0.091 (3)	−0.031 (2)	0.0150 (19)
C16	0.181 (4)	0.0621 (17)	0.103 (2)	−0.056 (2)	−0.044 (3)	−0.0020 (16)
C17	0.112 (2)	0.0597 (14)	0.0803 (17)	−0.0263 (14)	−0.0239 (15)	−0.0062 (13)
N1	0.0610 (9)	0.0469 (9)	0.0518 (9)	−0.0244 (7)	−0.0209 (7)	0.0028 (7)
N2	0.0782 (12)	0.0510 (10)	0.0617 (11)	−0.0349 (9)	−0.0330 (9)	0.0086 (8)
O1	0.0783 (10)	0.0512 (8)	0.0576 (8)	−0.0312 (7)	−0.0233 (7)	−0.0019 (7)
O2	0.1046 (13)	0.0593 (9)	0.0791 (11)	−0.0429 (9)	−0.0219 (9)	0.0115 (8)
S1	0.0601 (3)	0.0662 (3)	0.0720 (4)	−0.0300 (3)	−0.0214 (3)	−0.0055 (3)

### Geometric parameters (Å, º)

**Table:**

C1'—O1'	1.356 (2)	C1—O1	1.351 (2)
C1'—C6'	1.396 (3)	C1—C2	1.389 (3)
C1'—C2'	1.397 (3)	C1—C6	1.397 (3)
C2'—O2'	1.369 (2)	C2—O2	1.367 (3)
C2'—C3'	1.379 (3)	C2—C3	1.374 (3)
C3'—C4'	1.382 (3)	C3—C4	1.375 (4)
C3'—H3'	0.9300	C3—H3	0.9300
C4'—C5'	1.365 (3)	C4—C5	1.360 (3)
C4'—H4'	0.9300	C4—H4	0.9300
C5'—C6'	1.402 (3)	C5—C6	1.391 (3)
C5'—H5'	0.9300	C5—H5	0.9300
C6'—C8'	1.454 (3)	C6—C8	1.444 (3)
C7'—O2'	1.413 (3)	C7—O2	1.426 (3)
C7'—H7'1	0.9600	C7—H7A	0.9600
C7'—H7'2	0.9600	C7—H7B	0.9600
C7'—H7'3	0.9600	C7—H7C	0.9600
C8'—N1'	1.270 (2)	C8—N1	1.268 (2)
C8'—H8'	0.9300	C8—H8	0.9300
C9'—N3'	1.347 (3)	C9—N3	1.352 (2)
C9'—N2'	1.364 (3)	C9—N2	1.361 (2)
C9'—S1'	1.657 (2)	C9—S1	1.666 (2)
N3'—C12'	1.431 (3)	N3—C12	1.441 (3)
N3'—C11'	1.466 (3)	N3—C11	1.463 (3)
C11'—H11A	0.9600	C11—H11D	0.9600
C11'—H11B	0.9600	C11—H11E	0.9600
C11'—H11C	0.9600	C11—H11F	0.9600
C12'—C17'	1.366 (4)	C12—C17	1.372 (3)
C12'—C13'	1.375 (4)	C12—C13	1.373 (3)
C13'—C14'	1.382 (5)	C13—C14	1.388 (4)
C13'—H13'	0.9300	C13—H13	0.9300
C14'—C15'	1.363 (6)	C14—C15	1.358 (5)
C14'—H14'	0.9300	C14—H14	0.9300
C15'—C16'	1.348 (7)	C15—C16	1.368 (5)
C15'—H15'	0.9300	C15—H15	0.9300
C16'—C17'	1.389 (6)	C16—C17	1.386 (4)
C16'—H16'	0.9300	C16—H16	0.9300
C17'—H17'	0.9300	C17—H17	0.9300
N1'—N2'	1.372 (2)	N1—N2	1.364 (2)
N2'—H2'	0.83 (2)	N2—H2	0.80 (2)
O1'—H1'	0.79 (3)	O1—H1	0.87 (3)
			
O1'—C1'—C6'	123.80 (17)	O1—C1—C2	117.42 (17)
O1'—C1'—C2'	116.83 (16)	O1—C1—C6	123.16 (17)
C6'—C1'—C2'	119.37 (16)	C2—C1—C6	119.40 (17)
O2'—C2'—C3'	125.16 (18)	O2—C2—C3	124.7 (2)
O2'—C2'—C1'	114.36 (16)	O2—C2—C1	114.85 (18)
C3'—C2'—C1'	120.48 (18)	C3—C2—C1	120.4 (2)
C2'—C3'—C4'	119.92 (19)	C2—C3—C4	120.2 (2)
C2'—C3'—H3'	120.0	C2—C3—H3	119.9
C4'—C3'—H3'	120.0	C4—C3—H3	119.9
C5'—C4'—C3'	120.50 (19)	C5—C4—C3	119.9 (2)
C5'—C4'—H4'	119.7	C5—C4—H4	120.0
C3'—C4'—H4'	119.7	C3—C4—H4	120.0
C4'—C5'—C6'	120.62 (19)	C4—C5—C6	121.4 (2)
C4'—C5'—H5'	119.7	C4—C5—H5	119.3
C6'—C5'—H5'	119.7	C6—C5—H5	119.3
C1'—C6'—C5'	119.10 (17)	C5—C6—C1	118.64 (18)
C1'—C6'—C8'	121.69 (16)	C5—C6—C8	119.08 (18)
C5'—C6'—C8'	119.21 (17)	C1—C6—C8	122.27 (16)
O2'—C7'—H7'1	109.5	O2—C7—H7A	109.5
O2'—C7'—H7'2	109.5	O2—C7—H7B	109.5
H7'1—C7'—H7'2	109.5	H7A—C7—H7B	109.5
O2'—C7'—H7'3	109.5	O2—C7—H7C	109.5
H7'1—C7'—H7'3	109.5	H7A—C7—H7C	109.5
H7'2—C7'—H7'3	109.5	H7B—C7—H7C	109.5
N1'—C8'—C6'	119.62 (18)	N1—C8—C6	119.82 (17)
N1'—C8'—H8'	120.2	N1—C8—H8	120.1
C6'—C8'—H8'	120.2	C6—C8—H8	120.1
N3'—C9'—N2'	114.31 (19)	N3—C9—N2	114.93 (17)
N3'—C9'—S1'	123.28 (16)	N3—C9—S1	123.36 (14)
N2'—C9'—S1'	122.41 (16)	N2—C9—S1	121.71 (15)
C9'—N3'—C12'	122.44 (18)	C9—N3—C12	121.84 (16)
C9'—N3'—C11'	120.9 (2)	C9—N3—C11	120.89 (18)
C12'—N3'—C11'	116.4 (2)	C12—N3—C11	117.18 (17)
N3'—C11'—H11A	109.5	N3—C11—H11D	109.5
N3'—C11'—H11B	109.5	N3—C11—H11E	109.5
H11A—C11'—H11B	109.5	H11D—C11—H11E	109.5
N3'—C11'—H11C	109.5	N3—C11—H11F	109.5
H11A—C11'—H11C	109.5	H11D—C11—H11F	109.5
H11B—C11'—H11C	109.5	H11E—C11—H11F	109.5
C17'—C12'—C13'	119.9 (3)	C17—C12—C13	120.5 (2)
C17'—C12'—N3'	120.2 (3)	C17—C12—N3	119.8 (2)
C13'—C12'—N3'	119.9 (2)	C13—C12—N3	119.7 (2)
C12'—C13'—C14'	121.2 (3)	C12—C13—C14	120.1 (3)
C12'—C13'—H13'	119.4	C12—C13—H13	120.0
C14'—C13'—H13'	119.4	C14—C13—H13	120.0
C15'—C14'—C13'	118.1 (5)	C15—C14—C13	119.0 (3)
C15'—C14'—H14'	121.0	C15—C14—H14	120.5
C13'—C14'—H14'	121.0	C13—C14—H14	120.5
C16'—C15'—C14'	121.5 (5)	C14—C15—C16	121.4 (3)
C16'—C15'—H15'	119.3	C14—C15—H15	119.3
C14'—C15'—H15'	119.3	C16—C15—H15	119.3
C15'—C16'—C17'	120.7 (4)	C15—C16—C17	119.9 (3)
C15'—C16'—H16'	119.6	C15—C16—H16	120.1
C17'—C16'—H16'	119.6	C17—C16—H16	120.1
C12'—C17'—C16'	118.7 (4)	C12—C17—C16	119.1 (3)
C12'—C17'—H17'	120.7	C12—C17—H17	120.4
C16'—C17'—H17'	120.7	C16—C17—H17	120.4
C8'—N1'—N2'	118.72 (17)	C8—N1—N2	119.19 (16)
C9'—N2'—N1'	118.18 (18)	C9—N2—N1	117.98 (17)
C9'—N2'—H2'	123.2 (15)	C9—N2—H2	122.1 (15)
N1'—N2'—H2'	117.9 (15)	N1—N2—H2	119.2 (15)
C1'—O1'—H1'	106 (2)	C1—O1—H1	108.0 (17)
C2'—O2'—C7'	117.91 (17)	C2—O2—C7	117.5 (2)
			
O1'—C1'—C2'—O2'	−0.4 (2)	O1—C1—C2—O2	−0.3 (3)
C6'—C1'—C2'—O2'	179.33 (16)	C6—C1—C2—O2	−178.80 (18)
O1'—C1'—C2'—C3'	179.63 (18)	O1—C1—C2—C3	180.0 (2)
C6'—C1'—C2'—C3'	−0.6 (3)	C6—C1—C2—C3	1.5 (3)
O2'—C2'—C3'—C4'	−179.5 (2)	O2—C2—C3—C4	−179.7 (3)
C1'—C2'—C3'—C4'	0.4 (3)	C1—C2—C3—C4	0.0 (4)
C2'—C3'—C4'—C5'	0.4 (3)	C2—C3—C4—C5	−0.9 (5)
C3'—C4'—C5'—C6'	−1.0 (3)	C3—C4—C5—C6	0.3 (5)
O1'—C1'—C6'—C5'	179.77 (18)	C4—C5—C6—C1	1.1 (4)
C2'—C1'—C6'—C5'	0.0 (3)	C4—C5—C6—C8	−177.6 (3)
O1'—C1'—C6'—C8'	−0.2 (3)	O1—C1—C6—C5	179.6 (2)
C2'—C1'—C6'—C8'	−179.90 (17)	C2—C1—C6—C5	−2.0 (3)
C4'—C5'—C6'—C1'	0.8 (3)	O1—C1—C6—C8	−1.8 (3)
C4'—C5'—C6'—C8'	−179.30 (19)	C2—C1—C6—C8	176.68 (18)
C1'—C6'—C8'—N1'	0.9 (3)	C5—C6—C8—N1	179.5 (2)
C5'—C6'—C8'—N1'	−179.04 (19)	C1—C6—C8—N1	0.8 (3)
N2'—C9'—N3'—C12'	13.4 (3)	N2—C9—N3—C12	−13.3 (3)
S1'—C9'—N3'—C12'	−166.54 (19)	S1—C9—N3—C12	166.72 (16)
N2'—C9'—N3'—C11'	−173.3 (2)	N2—C9—N3—C11	170.3 (2)
S1'—C9'—N3'—C11'	6.7 (3)	S1—C9—N3—C11	−9.7 (3)
C9'—N3'—C12'—C17'	−104.5 (3)	C9—N3—C12—C17	113.3 (2)
C11'—N3'—C12'—C17'	81.9 (3)	C11—N3—C12—C17	−70.2 (3)
C9'—N3'—C12'—C13'	76.3 (3)	C9—N3—C12—C13	−69.1 (3)
C11'—N3'—C12'—C13'	−97.2 (3)	C11—N3—C12—C13	107.5 (2)
C17'—C12'—C13'—C14'	−1.1 (4)	C17—C12—C13—C14	−0.4 (4)
N3'—C12'—C13'—C14'	178.1 (3)	N3—C12—C13—C14	−178.0 (2)
C12'—C13'—C14'—C15'	−0.3 (5)	C12—C13—C14—C15	0.1 (4)
C13'—C14'—C15'—C16'	1.8 (7)	C13—C14—C15—C16	0.6 (5)
C14'—C15'—C16'—C17'	−1.9 (8)	C14—C15—C16—C17	−1.0 (5)
C13'—C12'—C17'—C16'	1.0 (5)	C13—C12—C17—C16	0.0 (4)
N3'—C12'—C17'—C16'	−178.1 (3)	N3—C12—C17—C16	177.6 (2)
C15'—C16'—C17'—C12'	0.4 (7)	C15—C16—C17—C12	0.7 (4)
C6'—C8'—N1'—N2'	−178.57 (18)	C6—C8—N1—N2	−178.81 (18)
N3'—C9'—N2'—N1'	−173.90 (19)	N3—C9—N2—N1	177.54 (17)
S1'—C9'—N2'—N1'	6.0 (3)	S1—C9—N2—N1	−2.4 (3)
C8'—N1'—N2'—C9'	−162.8 (2)	C8—N1—N2—C9	161.71 (19)
C3'—C2'—O2'—C7'	4.8 (3)	C3—C2—O2—C7	−11.3 (4)
C1'—C2'—O2'—C7'	−175.1 (2)	C1—C2—O2—C7	169.0 (2)

### Hydrogen-bond geometry (Å, º)

**Table:**

*D*—H···*A*	*D*—H	H···*A*	*D*···*A*	*D*—H···*A*
O1—H1···N1	0.87 (2)	1.85 (3)	2.611 (2)	146 (3)
O1′—H1′···N1′	0.80 (3)	1.89 (2)	2.609 (2)	149 (3)
N2—H2···O1′^i^	0.80 (2)	2.59 (2)	3.341 (2)	157 (2)
N2—H2···O2′^i^	0.80 (2)	2.52 (2)	3.081 (3)	128 (2)
N2′—H2′···O1	0.83 (2)	2.51 (2)	3.282 (3)	156.1 (19)
N2′—H2′···O2	0.83 (2)	2.62 (2)	3.214 (3)	130.0 (19)
C8—H8···O2′^i^	0.93	2.52	3.085 (3)	120

Symmetry code: (i) *x*, *y*−1, *z*.
